# Report of Trustees of Illinois Hospital for the Insane

**Published:** 1849

**Authors:** 


### ARTICLE IV.

*First Biennial Report of the Trustees of the Illinois State Hospital for the Insane.* (To the General Assembly.)

This institution, now in process of erection situated one mile south of Jacksonville, Illinois, was commenced in the summer of 1847, and now has the foundation permanently and, we are informed, substantially laid. The document before us is made up principally of accounts in reference to procuring information and business details.

The plan of the building at Indianapolis, with some slight alterations by the board was adopted; but as the proportions of the building with a high foundation such as has been laid, will be improved by it, and the probable wants of the state for a number of years, met, it is hoped that instead of spoiling a whole story by making it low to be called a "basement," the trustees will so far alter the plan as to reduce it to three stories, and have no basement. This may be done with more propriety, as there is a cellar under the whole building already. All considerations of economy should be secondary to the arrangement of such a building for the comfort and cure of patients, and the convenience of surveillance; and no expense should be spared to make it right while being constructed, as blunders here are irremediable; but by this proposed change, a very heavy expense in making rooms not very suitable for patients would be saved, and the institution would be ready for the admission of patients at least one year earlier than otherwise; certainly points of much importance.

Dr. James M. Higgins was elected superintendent on the 12th of August last. His term of office is, by law, ten years, but he does not enter upon the duties until this spring or at the pleasure of the board as to time.

A code of by-laws for the government of the institution while being built is published, which we think very good.

The whole amount expended up to the date of the report for materials, work, etc., as shown by the treasurer's statement, is $13,121 54.

It is to be hoped that no delay will be allowed to prevent a speedy completion of at least a part of the building so as to allow of the reception and treatment of patients. E.

